# Biomedical Waste Management by Using Nanophotocatalysts: The Need for New Options

**DOI:** 10.3390/ma13163511

**Published:** 2020-08-09

**Authors:** Sara Hooshmand, Saeid Kargozar, Ahmad Ghorbani, Majid Darroudi, Meysam Keshavarz, Francesco Baino, Hae-Won Kim

**Affiliations:** 1 Pharmacological Research Center of Medicinal Plants, Mashhad University of Medical Sciences, Mashhad 917794-8564, Iran; s_hooshmand@yahoo.com (S.H.); ghorbania@mums.ac.ir (A.G.); 2 Tissue Engineering Research Group (TERG), Department of Anatomy and Cell Biology, Mashhad University of Medical Sciences, Mashhad 917794-8564, Iran; 3 Nuclear Medicine Research Center, Mashhad University of Medical Sciences, Mashhad 917794-8564, Iran; Darroudim@mums.ac.ir; 4 Hamlyn Centre, Imperial College London, Bessemer Building, South Kensington Campus, Exhibition Road, Kensington, London SW7 2AZ, UK; m.keshavarz@imperial.ac.uk; 5 Institute of Materials Physics and Engineering, Applied Science and Technology Department, Politecnico di Torino, Corso Duca degli Abruzzi 24, 10129 Torino, Italy; 6 Institute of Tissue Regeneration Engineering (ITREN), Dankook University, Cheonan 31116, Korea; kimhw@dku.edu; 7 Department of Nanobiomedical Science & BK21 PLUS NBM Global Research Center for Regenerative Medicine, Dankook University, Cheonan 31116, Korea; 8 Department of Biomaterials Science, School of Dentistry, Dankook University, Cheonan 31116, Korea; 9 UCL Eastman-Korea Dental Medicine Innovation Centre, Dankook University, Cheonan 31116, Korea

**Keywords:** nanophotocatalysts, biomedical waste management, waste treatment technologies, green chemistry

## Abstract

Biomedical waste management is getting significant consideration among treatment technologies, since insufficient management can cause danger to medicinal service specialists, patients, and their environmental conditions. The improvement of waste administration protocols, plans, and policies are surveyed, despite setting up training programs on legitimate waste administration for all healthcare service staff. Most biomedical waste substances do not degrade in the environment, and may also not be thoroughly removed through treatment processes. Therefore, the long-lasting persistence of biomedical waste can effectively have adverse impact on wildlife and human beings, as well. Hence, photocatalysis is gaining increasing attention for eradication of pollutants and for improving the safety and clearness of the environment due to its great potential as a green and eco-friendly process. In this regard, nanostructured photocatalysts, in contrast to their regular counterparts, exhibit significant attributes such as non-toxicity, low cost and higher absorption efficiency in a wider range of the solar spectrum, making them the best candidate to employ for photodegradation. Due to these unique properties of nanophotocatalysts for biomedical waste management, we aim to critically evaluate various aspects of these materials in the present review and highlight their importance in healthcare service settings.

## 1. Introduction

Biomedical waste management has recently risen as one of the major challenges that developing countries are confronting. The amount of biomedical waste produced has considerably increased as the worldwide populace has expanded, and accessible assets are not sufficient to deal with it [1]. Disposal and post treatment of the waste produced in the healthcare system may indirectly cause health hazardous through the release of pathogens and toxic pollutants into the environment. Discarding the untreated healthcare wastes in landfills, if the landfill is not properly constructed, can lead to the contamination of surface, drinking and ground water resources. Additionally, treatment of healthcare wastes with chemical disinfectants can result in the release of chemical substances into the environment if those substances are not handled, stored and disposed of in an environmentally sound manner. Incineration of waste has also been widely practiced; however, insufficient incineration or the incineration of inappropriate materials pollute the air and generate ash residue. Lack of knowledge about the health-related hazards of healthcare waste, inadequate training in proper waste management, absence of waste management and sufficient disposal systems, insufficient financial and human resources and the low priority given to the topic are the most common problems associated with healthcare waste. Many countries either do not have appropriate regulations, or do not enforce them.

Successful and efficient management of biomedical waste requires the use of different treatment practices and techniques, such as incineration, autoclave, hydroclave, and microwave treatments [2]. It is essential that every single applied innovation assures both the environment and public health protection [3]. Waste management and waste engineering advances have turned out to be extremely vital because of the significant increase in rate and diversity, both in the quality and quantity of the waste that is being produced every day, so using the most financially plausible strategies has become even more crucial than before [4]. Since waste products cannot be completely eradicated, the choice of waste treatment has become specifically important as its management.

The other commonly used methods to treat biomedical waste are mechanical treatments such as granulation, pulverization, shredding, grinding, mixing, agitation, and crushing. This type of treatment has the advantage of reducing the bulk volume of the waste materials by 60 percent or more. Although mechanical treatment does not remove the pathogens or disinfect equipment, it reduces the waste volume to facilitate further treatment or disposal. Equipment involved in mechanical treatment includes but is not limited to crushers, millers, shattering machines and splinterers. These treatment methods can alter the appearance of the waste, which can be useful in reducing the psychological impact of the waste on human observers. In addition to reducing the volume of bulk disposal, mechanical treatment can increase the surface area of the solid pieces before subsequent chemical or heat treatment.

Chemical disinfection, such as through the use of chlorine compounds, has been widely used to eliminate the microorganisms in medical waste, as well as oxidizing hazardous chemical constituents. For instance, chlorine bleach has been used to disinfect swimming pools and reduce the risk of disease transmission. Another example of chemical disinfectant compound is ethylene oxide treatment, which is used to disinfect materials and sometimes to treatment of medical waste. Ethylene oxide (EtO) treatment is used to sterilize the equipment that will be frequently used. This disinfectant chemical is not cost effective for use on equipment or treatment of waste that will be disposed of in a landfill. EtO gas can be used to kill microorganisms and disinfect products during packaging processes.

Microwave radiation has recently been employed to treat wastewater sludge and to generate heat for treating medical waste. This method of waste treatment can be employed either on-site or mobile by using treatment vehicles. To enhance the efficacy of the microwave treatment and reduce the volume of the final product, the waste will go through a shredding process first. In the case of using dry waste, the waste is wetted with water and then introduced into the microwave chamber, as this method of disinfection is effective only when the waste is damp. Therefore, the microwave treatment units are usually supplied with a humidifier. Although the whole disinfection time is determined by the manufacturer and experience of the operators, approximately 20 min per each batch is required. Microwave should not be confused with irradiation such as gamma rays (from radioactive elements) or electrons, as these two methods are completely different.

Gamma irradiation is a means of sterilization by exposing waste to gamma rays, as it breaks down bacterial DNA. To generate gamma rays, radioactive isotopes of cobalt are employed, this is the same radiation source used for the radiation treatment of cancer. However, in cancer treatment, the radiation is intended to kill the malignant cells, whereas to sterilize equipment or treat waste, pathogens are targeted. In contrast, the ultraviolet (UV) radiation used to treat wastewater is not capable of killing microbes so much as it is able to break down chemicals. The efficiency of irradiation as a means of sterilization is highly dependent on the total energy delivered, but even then, this method of treatment suffers from the shadowing effect, which means that waste surfaces facing the radiation source are more sterile than the waste on the shaded side. Therefore, waste with odd shapes, and the sides of contaminated surfaces facing away from the cobalt source, may not be adequately exposed to the radiation. Heat treatment, by contrast, brings every piece of waste to an adequate temperature for sterilization, if done properly.

Although vitrification—the means production of glass—has rarely been used, it could be an effective treatment for medical waste. The high temperature kills pathogens and some combustible material via burning or pyrolysis, which results in an off-gas. The remaining by-product is encapsulated in glass, which has a very low diffusivity. However, this method of disinfection might become dangerous if significant quantities of the encapsulated hazardous material leach out of the glass. Ultimately, the vitrified waste can be disposed of in a landfill with confidence. Despite the development of plasma treatment as an alternative to incineration for medical waste treatment, it has not been widely implemented [5].

To decrease the expense of waste treatment through cost-effective strategies, various methods based on the exploitation of sunlight have been proposed for both solid and liquid waste management [6]. Among these, photocatalysis is a remarkable technique with a variety of applications, including the debasement of different contaminations in wastewater [7], antibacterial functions [8], cleansing of air [9], and generation of hydrogen [10]. The photocatalytic procedure is attracting more focus in the field of ecological and environmental safety, as there is a need to achieve the utmost degradation of contaminants attainable under states of mild pressure and temperature. The significant highlight of these procedures is the incorporation of cost-effective near-UV (from 400 nm down to 300 nm) light, with sunlight as an alternative source of irradiation. The term photocatalysis refers to a chemical reaction using light in the presence of a catalyst that assimilates light quanta and is associated with the chemical transformations of the reactants [11].

The optimal treatment system depends on many factors, such local conditions, availability of resources including technical expertise, waste characteristics and volume, relevant national regulations and safety requirements, technical requirements for installation, operation and maintenance of the treatment system, environmental factors and cost considerations. Nonetheless, waste management systems can be changed and improved only within the financial and technical capacity of a given health-care system, which may then require making small decisions towards an incremental improvement, as well as planning for the attainment of long-term improvements, once certain conditions have been met.

Nanophotocatalysts have been widely used to treat waste in the field of environmental and ecological safety, as they have numerous benefits, such as those of low cost, superb stability, high photocatalytic activity, innocuousness to humans, etc. [12]. Different methods, including ion exchange microorganisms and adsorption, have been used to treat sewage. However, these methods are restricted due to their complex technology, high cost, risk of second contamination, and poor degradation effectiveness [13]. Compared to other methods for biomedical waste management, nanophotocatalysts are considered one of the most particular strategies with respect to energy consumption, environmental and ecological issues. The advantages of the photocatalysis strategy are as follows [14]: (a) photocatalysis offers a decent alternative to the traditional energy-concentrated treatment techniques (e.g., ultrafiltration and reverse osmosis) with the capability of using pollution-free and renewable solar energy; (b) it prompts the creation of innocuous products, in contrast to traditional treatment methods in which pollutants only transfer from one phase to another; (c) the procedure can be used for the decimation of an assortment of risky and hazardous compounds in various wastewater streams; (d) it requires less chemical input and can be operated under mild reaction conditions with modest reaction time; (e) minimum generation of secondary waste; (f) it can also be applied to a solid phase (soil), gaseous phase (hydrogen generation), and aqueous treatments. The dependency of the photocatalytic activity on the following criteria has hampered their application [15]: (a) charge separation; (b) interfacial charge transfer needs to be improved; (c) charge carrier recombination can be inhibited.

Although approaches to removing pollutants based on nanostructured catalytic membranes, nanosorbents and nanophotocatalyst are eco-friendly and efficient, in order to purify the waste, they require more energy and sufficient investment. There are many challenges involved in biomedical waste treatment; some precautions are required to keep hazardous waste away from ecological and health issues. New modern equipment for waste treatment is required to be flexible, low cost and efficient for commercialization purposes. Recently, with advancements in nanomaterials such as nanophotocatalysts, nanomotors, nanomembranes, nanosorbents and imprinted polymers, the decontamination of biomedical waste has been effectively revolutionized.

However, there has not been a systematic characterization of the risk and hazards related to nanomaterials, and there is a lack of safety regulations for using such catalysts. Overall, nearly all nanocatalysts have toxic effects both in vitro and in vivo at certain concentrations. For instance, ROS generation and cell signaling perturbations are widely accepted causes of nanotoxicity. Furthermore, the toxicity of nanoparticles is also determined by factors such as particle size and surface functionalization [16]. Although to some degree, the toxicity and adverse effects of commonly used nanocatalysts have been realized, a comprehensive investigation is still required [17].

**Figure 1:**
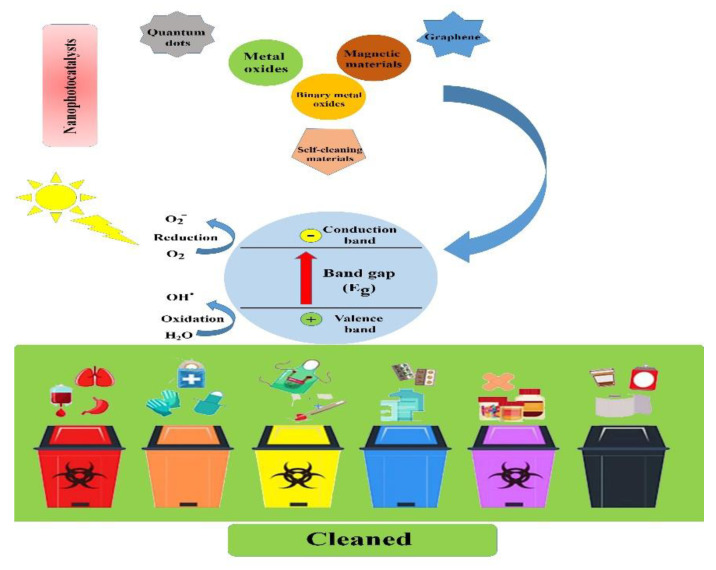
Schematic illustration showing the main types of nanophotocatalysts and the process by which biomedical and biological waste materials are cleaned.

This review aims to introduce different types of nanophotocatalysts and provide the main principles, mechanisms, and operating parameters of the photocatalysis process and emphasize its importance in biomedical waste management in detail (see Figure 1).

## 2. Fundamentals and Mechanism of Photocatalytic Reactions

**Figure 2:**
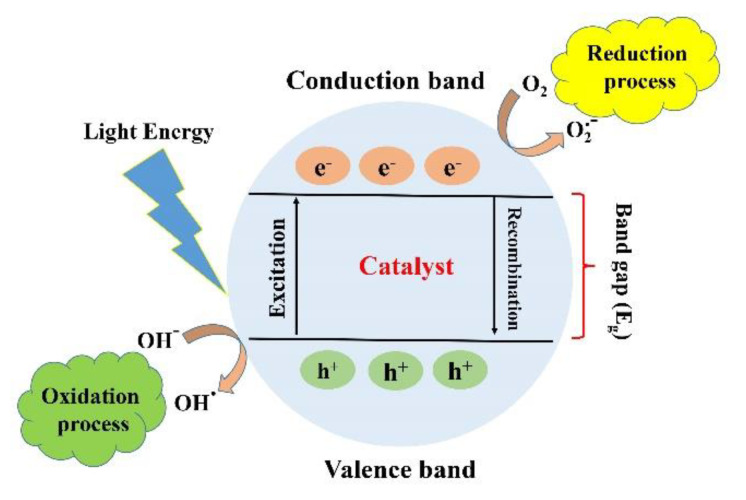
Schematic illustration of the photocatalytic mechanism. With permission from [18].

Photocatalysis is defined as a series of chemical reactions that is usually initiated by electromagnetic irradiation. The photocatalysis process can be divided into two main stages of reduction and oxidation. When a material is irradiated with photons with energy equal to or higher than its bandgap, the excited electrons in the conduction band (CB) will jump to the valence band (VB) through the bandgap leaving positive holes, which is called reduction. As a result, the generated electrons and holes lead to the formation of reactive oxygen spices (ROS) such as O_2_ and OH (oxidation). The kind of ROS depends on the type of material and irradiated photons. A conventional photocatalysis procedure is demonstrated in Figure 2. The formation of ROS is the significant outcome of photocatalysis that can lead to various effects such as degradation of dye and antibacterial activity. The efficiency of the photocatalysis process can be estimated by its impact on its surroundings, such as degradation, reduction, adoption, or antibacterial activity. The conventional method for assessing the efficiency of the photocatalytic process is to compare between the initial concentrations of the undesirable substances with the concentration of these substances after the photocatalytic reactions. The efficiency of photocatalytic activity is defined by the yield of electrons and holes created. The recombination of the electron–hole pair is one of the main factors that diminishes the efficiency of the photocatalytic activity.

In the photocatalytic process, the catalyst and light are simultaneously used to accelerate a chemical reaction. Thus, photocatalysis can be defined as the catalysis-driven speed-up of a light-actuated reaction. Photocatalysts are categorized into two classifications: hetero- and homogeneous procedures [19]. When comparing the two processes, the heterogeneous procedure is, in fact, a feasible strategy that can be exploited to reduce a variety of wastewater pollutions, and which has some benefits over other competing methods [20]. This procedure does not suffer from waste disposal problems, but offers complete mineralization and low cost, with the only requirement being mild pressure and temperature conditions. Homogeneous photocatalysis is mostly used with metal compounds as catalysts (e.g., copper, chromium, and iron transition metal complexes). In this procedure, thermal and photon conditions are used concurrently to generate hydroxyl radicals from the higher oxidation state of metal ion complexes. Hence, the reaction between these hydroxyl radicals with organic matters deactivates toxic compounds [21]. Photocatalytic response fundamentally relies upon the catalyst and energy of the light (photon). Semiconducting materials are generally used as catalysts for sensitization; the light irradiation stimulates the redox process because of their characteristic electron structure by a vacant conduction band and a filled valence band [22].

The influence of different factors on photocatalytic efficiency has been investigated and further optimized using the response surface methodology (RSM). The RSM is a combination of both statistical and mathematical methods to optimize a complicated process. To optimize the process more accurately, this statistical design of experiments considers the interaction effects between the studied parameters, and can determine the combination of levels. Furthermore, a central composite design (CCD) based on RSM can be successfully applied in the optimization of photodegradation of various organics [23].

Since the adsorption of pollutants on the surface of solids plays a critical role in heterogeneous photocatalysis, the effect of adsorption on photocatalytic process has been investigated. Simultaneous adsorption and photocatalytic processes has been used for degradation of phenol pollutants with noticeable recyclability and stability of the photocatalyst [24], bacterial disinfection [25], photocatalytic water purification [26,27,28], and photocatalytic mineralization of phenolic compounds.

## 3. Types and Characteristics of Nanophotocatalysts

Nano-sized photocatalyst particles demonstrate a significantly intensified reactivity compared to larger particles or bulk materials due to their large surface area [29,30]. Novel nanophotocatalysts have been developed with the ability to exploit solar energy to synthesize organic compounds under controlled conditions [31]. Nanophotocatalysts are mainly classified as surface plasmon resonance-mediated, metal-organic charge-transfer-based, and semiconductor-based nanophotocatalysts, capable of driving various organic reactions photocatalytically. Accordingly, they can be categorized as graphene semiconductors, composites of two semiconductors, core–shell composites, non-metal-doped semiconductor materials, and metal-modified semiconductors.

Metal-free catalysis, such as with graphitic carbon nitride nanocomposites, has also been used in photodegradation of aqueous phase organic pollutants [32]; likewise, polymeric graphitic carbon nitride-based Z-scheme photocatalytic systems, magnetic graphitic carbon nitride photocatalyst, and carbon quantum dot-supported graphitic carbon nitride have been respectively employed for sustainable photocatalytic water purification [28], degradation of oxytetracycline antibiotic [33], and photodegradation of 2,4-dinitrophenol [34].

Among different kinds of photocatalysts, metal oxide semiconductors, including TiO_2_, ZnO, α-Fe_2_O_3_, and WO_3_ are the most suitable ones, since they are photocorrosion resistant and have a wide band gap energy. TiO_2_ is currently used as the most efficient photocatalyst, and is widely applied in wastewater treatment, since it can promote the oxidation of organic compounds while being thermally stable, non-toxic, cost-effective, and chemically and biologically inert. Structural and surface properties, including surface area, porosity, crystal composition, particle size distribution, and band gap energy, are able to affect the photocatalytic activity of the catalyst [35]. The following characteristics typically represent nanophotocatalysts with specific favorable benefits over bulk materials [36]. First, their large surface area to volume ratio results in a high particle fraction, and subsequently a high division of active sites on the catalyst surface. Second, their valence band–conduction band energy gap strongly depends on the size of the nanoparticles [37].

Moreover, changing the size of the nanocatalyst makes it possible to adjust the absorbance wavelength. Additionally, the optical and electronic characteristics of the nanocatalyst can be modified by tuning their sizes [38]. Due to all these favorable properties, nanophotocatalysts have been employed in a wide range of reactions, for instance, in organic synthesis, splitting water to hydrogen fuel generation, inactivation of cancer cells, and dye degradation [39,40]. Primary studies on the development of nanophotocatalysts have mainly focused on their degradation capabilities of pollutants and dyes. Given these properties, there is an increasing interest in the use of nanophotocatalysts as catalysts for different organic reactions, providing options in green chemistry as alternatives to the regular techniques used in research laboratories and industry, which apply thermal energy to achieve the same goals [41].

The efficacy rate in photocatalysis methods is primarily dependent on the size, shape, crystal structure and surface area of the photocatalyst, as well as the morphology, which can also act as a significant factor affecting the final degradation throughput. The amount of catalyst is also directly proportional to the overall rate of photocatalytic reaction. Disinfection efficiency of the photocatalyst can be improved by an increase of its doses [42]. The pH of the solution is another effective factor, as it determines the photocatalyst surface charge properties. Furthermore, various pH values may impact the efficiency of the disinfection process, values ranging from 6.0 to 8.0 have shown the highest impact. Different studies have evidenced that the microorganisms have pH sensitivity at around 6.5 and 8.0, the range in which photocatalytic activity has been demonstrated to be excellent. This is due to the fact that as pH moves away from neutral, the effectiveness of the overall process declines when pH reaches 7 [43]. The optimum range of reaction temperature which primarily depends on the activation energies of the materials in the photocatalytic reaction [44], the light intensity, which mainly influences the degradation rate of photocatalytic reaction [45], and the nature and concentration of pollutants [42] can also affect the performance of nanophotocatalysts. Furthermore, inorganic ions such as iron, magnesium, copper, zinc, phosphate, bicarbonate, chloride, sulfate and nitrate can change the rate of photocatalytic degradation of the organic pollutants since they can be adsorbed onto the nanophotocatalyst surface [46].

## 4. Biomedical Applications of Nanophotocatalysts

**Figure 3:**
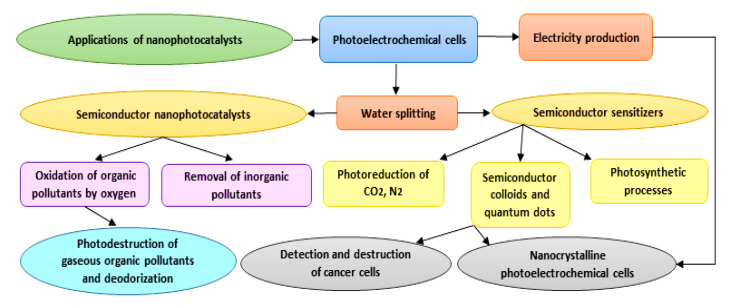
Diagram showing different applications proposed for nanophotocatalysts.

As photocatalysts have a superior capability in the deactivation of various destructive microorganisms, they could reasonably be used as alternatives to conventional techniques (e.g., chlorination), which can generate harmful and undesirable by-products [47]. Photocatalysis is a flexible and successful procedure that can be adopted in numerous cleansing applications in both air and water frameworks [48]. Furthermore, photocatalytic surfaces have been used on account of their self-sanitizing attributes. Photocatalytic applications have been recently developed specifically in the contexts of environmental health and indoor air, plant protection, effluents, wastewater and drinking water disinfection, dye removal, the pharmaceutical and food industries, laboratories and hospitals, and biological and medical applications. Due to the low energy consumption and feasible accessibility to solar energy and decreased treatment time, the overall cost for photodegradation of hazardous compounds and pollutants is lower, and hence beneficial [49]. Applications of nanophotocatalysts are summarized in Figure 3. With respect to the title of the study, we focus on biomedical applications of nanophotocatalysts in the following sections.

### 4.1. Laboratory and Hospital

#### 4.1.1. Basics and Fundamentals

**Figure 4:**
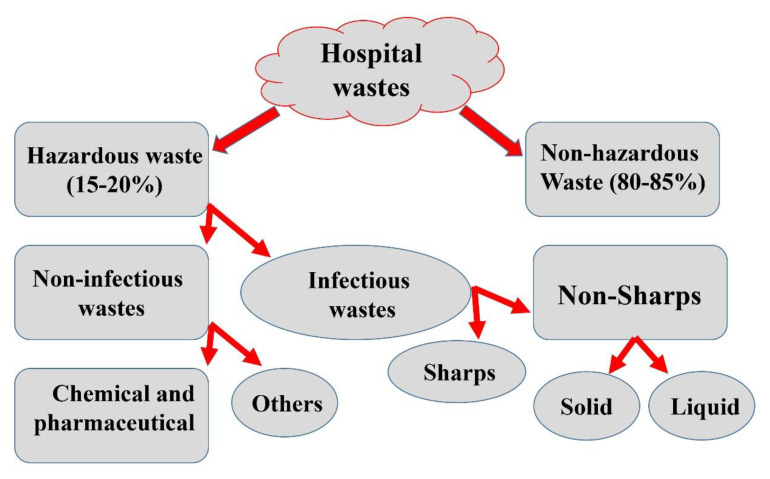
The schematic representation of different types of hospital waste.

Hospital waste can be described as a combination of both biological and non-biological waste that is discarded and not intended to be used again. Hospital and laboratory waste can be broadly defined into two categories: hazardous (risk waste) and non-hazardous (non-risk) waste (Figure 4). Risk waste includes sharps, pathological, pharmaceutical, chemical, and radioactive waste, whereas non-risk waste is equivalent to general domestic garbage and introduce no greater risk than normal home waste (e.g., paper, packaging, and food waste). A portion of hospital and laboratory waste management processes includes segregation, handling, transportation, disinfection, mutilation, storage, and final disposal as the foundation for essential progress towards scientifically safe waste management [50].

**Figure 5:**
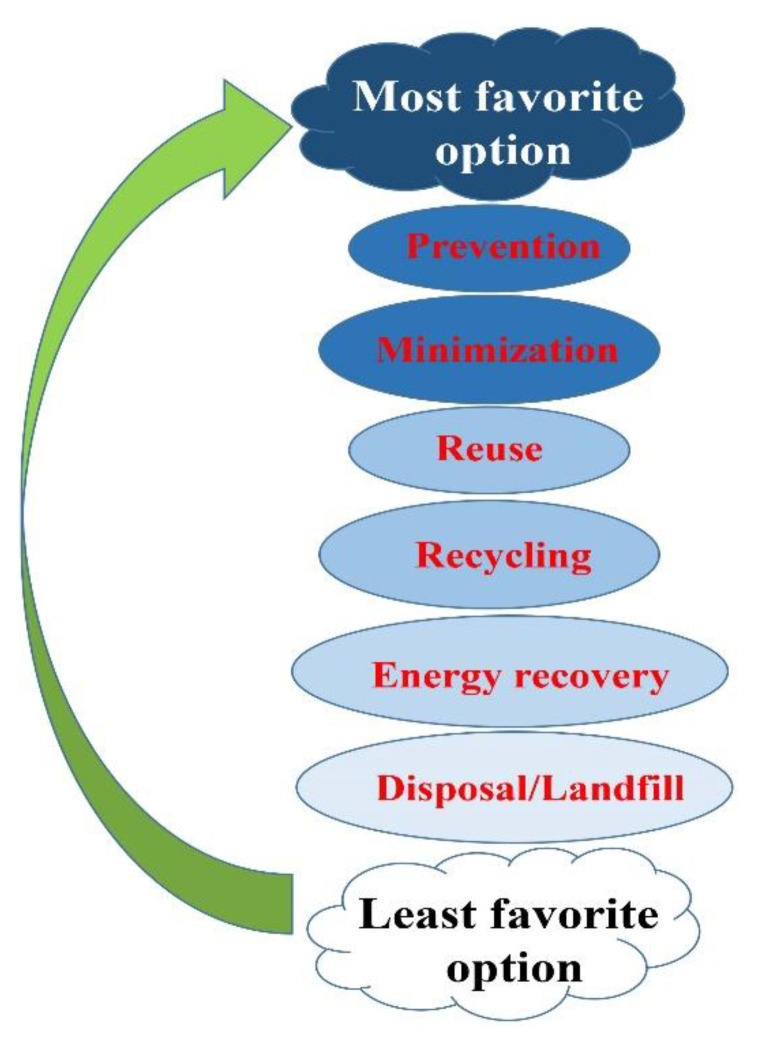
A schematic illustration of concepts of waste management. The hierarchy of management of wastes in order or preference, starting with prevention as the most favorable, down to disposal as the least favorable option.

There are two main steps involved in the waste disposal process: treatment and final disposal. In the treatment step, depending on the nature of the waste, procedures such as incineration, autoclaving, chemical disinfection, encapsulation, and microwave irradiation are usually applied. The disposed wastes are likely to wind up in landfill, buried inside the premises, discharged into the sewer, etc. Segregation (separation) has a key role in an efficient waste identification and management process. Sorting the waste based on the color of the container is the most proper method for identifying waste categories [51]. The last treatment applied to the wastes can be performed by autoclave, hydroclave, incineration, or microwave technologies. Waste disposed of in landfill must be properly designated and managed, despite being the cheapest and most readily available way. On the other hand, shredding involves all types of bulk plastic waste, including risk waste, which are first disinfected, and then cut into small pieces and converted into a compact form. This approach is cost effective, and is much cheaper than an incinerator, while causing comparatively no pollution. Autoclaving, however, as the last type of waste disposal is more expensive, but more promising than shredding. People who are exposed to hazardous hospital waste are potentially at higher risk—either those who handle the waste at any stage or are exposed as a consequence of careless management [52]. The most to least favorable options of waste management are demonstrated in Figure 5.

#### 4.1.2. Photocatalytic Point of View

**Figure 6:**
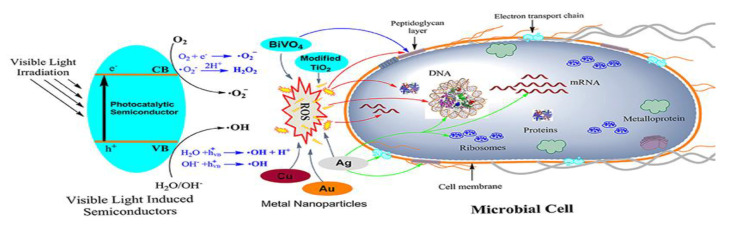
Schematic representation of the antimicrobial mechanisms proposed for different photocatalytic semiconductors. As shown on the left side, the photocatalytic semiconductor is activated via visible light. The main targets of ROSs generated by the semiconductors are indicated with red colored arrows, while the blue and green colored arrows indicate the target of BiVO_4_ and Ag nanoparticles, respectively. Reproduced from [55].

Photocatalysis is an efficient, affordable strategy for the decontamination and disinfection of hospital waste that applies ultraviolet rays or solar energy to disintegrate antibiotics and disinfect microbes from the waste at the point of origin (see Figure 6). In demanding cases such as medical care systems and especially in microbiological labs, frequent and thorough cleaning of surfaces is required to inhibit bacterial transmission and diminish the emergence of microscopic organisms (Table 1). Traditional sterilization strategies are time- and staff-consuming, and do not usually have long-term effectiveness. Additionally, the use of ultraviolet (UV) light instead of aggressive and dangerous chemicals can cause severe occupational health risks [53]. Titanium dioxide-coated surfaces can perform photocatalytic oxidation as an alternative to the conventional techniques of surface cleansing. The disinfection attributes of anchored titanium thin films on solid surfaces have been investigated in some previous studies [53,54]. The feasibility of this procedure for hygiene has been exhibited using bacteria, for example, *Staphylococcus aureus* (*S. aureus*), *Pseudomonas aeruginosa* (*P. aeruginosa*), *Enterococcus faecium* (*E. faecium*), and *Escherichia coli* (*E. coli*) [53].

Another example of self-disinfecting surface application is the inactivation of the deposited *E. coli* (ATCC8739) cells on membrane filters during fluorescent light irradiation [56]. In another study, a novel flame-assisted chemical vapor deposition (CVD) technique was used to test the antimicrobial activity of the stainless steel coated with TiO_2_ thin films on *E. coli* [57]. Due to the extensive applications of the self-sterilizing material stainless steel, because of corrosion resistance, has been used for sterilization of *Bacillus pumilus (B. pumilus)* and has shown higher photocatalytic activity compared to the glass substrates coated with self-sterilizing materials [58].

Surfaces coated with CuO-doped Titania photocatalysts were also assessed for their biocidal activity and synergistic impact of photocatalysis and lethality of copper to deactivate bacteriophage T4 and *E. coli* [59]. Nitrogen-doped TiO_2_ photocatalysts have been used owing to their visible light-induced bactericidal activity against human pathogens [60,70]. Visible light photocatalytic disinfection offers a continuous cleansing of surfaces that are constantly in contact with people, such as push buttons or door handles. This quality has been applied in the inactivation of *E. coli* using nitrogen/sulfur-co-doped Titania [61,62,63,71], which presents new disinfectant applications in public places that are consistently exposed to the transmission of pathogens (e.g., hospitals, schools, stations, hotels, airports, public toilets, and public transportation) [72,73].

In another study, prions, an infectious agent of a group of transmissible, fatal neurodegenerative disorders influencing both animals and humans, were inactivated using photocatalysts [48]. Photocatalytic oxidation, which is the cause of prion inactivation, can decrease the risk of spread, and has shown a significant effect on the pragmatic employment of this approach towards disinfection and cleansing of contaminated objects and surfaces, since these prions can be transmitted by ingestion of contaminated food or during medical treatments using contaminated surgical tools or biological materials. Controlling Legionnaire’s disease, which is related to the contamination of hot water distribution systems with *Legionella* bacteria, is another application of photocatalysts in a laboratory or hospital [64].

It has also been shown that TiO_2_/UV photocatalytic oxidation has the capacity to mineralize the four strains of *L. pneumophilia* serogroup 1 cells (strains 977, 1004, 1009, and ATCC 33153) in laboratory-scale surveys, which proves that this could be used as a reasonable procedure for the routine disinfection and sterilization in order to control Legionella bacteria species in the hot water systems of emergency clinics and hospitals (e.g., hyperchlorination and thermal eradication) [48]. The application of TiO_2_ as an effective treatment has also been reported to remove pharmaceutical contaminants from water with high efficiency. Due to their high photolysis sensitivity in aquatic systems, they have been used, for instance, to treat diclofenac [74,75], and triclosan [76].

### 4.2. Biological and Medical

#### 4.2.1. Categories and Conventional Methods

Biomedical waste refers to any type of waste generated during treatment, diagnosis, or research activities—testing biological products and the immunization of human beings or animals. Biomedical waste management has become a general medical issue, as it is not only a legal necessity, but also a social responsibility. An improvement of biomedical waste management starts with the reduction of the waste produced [77].

According to an earlier report, around half of the world’s population is in danger of incompatible biomedical waste management, which can impact both work and public places [78]. About 75% to 90% of total biomedical waste is general non-hazardous waste. The remaining 10% to 25% is dangerous and hazardous (including infectious waste, sharp waste, pathological waste, cytotoxic waste, pharmaceutical waste, liquid infectious waste, chemical waste, radioactive waste, and general health-care waste), which leads to a wide range of issues such as environmental and health risks. The main risks associated with biomedical waste include the microbial, systemic, and local infections caused by exposure to biomedical waste, among which pesticides, disinfectants, and mercury have multiple impacts in different ways, while inappropriate handling of sharps can lead to needle stick injuries and cause infections with blood-borne pathogens such as human immunodeficiency viruses (HIV), hepatitis B virus (HBV), and hepatitis C virus (HCV) [79].

The essential needs in biomedical waste management include reducing, waste storage and transport, recycling and reusing, expenses in the annual budget, storage management, separate chemical and pharmaceutical waste segregation, separate storage zones, and documentation related to biomedical waste management [80]. Waste treatment technologies can be summarized as thermal (autoclaves, steam, microwave, dry heat treatment technologies) [80,81], chemical processes (sodium hypochlorite (NaOCl, 1%–12%), chlorine dioxide, calcium hydroxide, glutaraldehyde and peracetic acid) [82], incineration [83], encapsulation and inertization, irradiation technologies, biological processes, membrane bioreactors [84], disinfection and sterilization, as well as emerging technologies such as alkaline hydrolysis, plasma pyrolysis, superheated steam, ozone and promession [85]. Other upcoming technologies for the destruction of biomedical waste include base-catalyzed decomposition, gas-phase chemical reduction, sodium reduction, supercritical water oxidation, verification, superheated steam reforming, Fe-tetra-amido macrocyclic ligand (TAML)/peroxide treatment (pharmaceutical waste), biodegradation (using mealworm or bacteria to eat plastics), mechanochemical treatment, sonic technology, electrochemical technologies, phytotechnology, and solvated electron technology [86].

#### 4.2.2. Photocatalytic Strategies

Photocatalytic processes are used in biomedical applications due to their disinfection abilities. It has been investigated that *Staphylococcus aureus* (*S. aureus*), a typical pathogenic bacterium in implant-related infection, shows photocatalytic activity using TiO_2_ film on titanium substrates and stainless steel [87]. TiO_2_ coatings have been used on bioimplants to apply photocatalysis for antibacterial purposes. The coatings exhibited a bactericidal impact upon UV irradiation, so the implementation of these photocatalytic-coated substrates are a valuable system for controlling infections related to biomedical implants [57,65,66]. As recently suggested for air purification systems, photocatalysis can be used to extract dangerous airborne biological risks, such as Anthrax, for example. Therefore, it is a versatile procedure for circumventing the spread of airborne biological threats and preventing bioterror dangers [67]. Reducing the number of bacteria and preventing their dissemination is essential and can be achieved through disinfection of surfaces in microbiological laboratories, food processing plants, veterinary medicine clinics, and hospitals. Traditional disinfection methods are generally time-consuming and tedious, and not usually sufficient (e.g., cleansing with chemical disinfectants) [88]. A significant alternative to conventional disinfection methods is the photocatalytic process by coating the surfaces with a thin layer of metal oxide nanostructures [89]. The high bactericidal property has added value for the practical application of photocatalysis in the treatment of microorganisms (e.g., *Pseudomonas aeruginosa*, *E. coli*, *Enterococcus faecium*, and *S. aureus*) [68,90], and plays a crucial role in public health protection [69,91,92].

##### Metal Oxides

The development of nanostructured metal oxides, semiconducting oxides, conducting oxides, composites, and polymers have been broadly investigated for quantification and detection of different hazardous biochemicals and chemicals [93]. It has been shown that metal oxides can photo-oxidize a wide range of organic substances such as alkenes, alkanes, surfactants, aromatics and pesticides [94]. Several metal oxides, including TiO_2_, ZnO, α-Fe_2_O_3_, MoO_3_, WO_3_, ZrO_2_ and SnO_2_ can be applied as photocatalysts, which are of great interest in current studies [95].

1. Titanium Dioxide (TiO_2_)

TiO_2_ is desirable for photocatalysis due to its stability, inertness, and low cost. It is also recyclable and self-regenerating. One of the most important industrial applications of TiO_2_-based photocatalysts is the degradation of expired drugs and pharmaceutical compounds [96], dyes in textile industries [97], toxic compounds spills (e.g., pesticides) [98], natural toxins (e.g., cyanobacterial toxin microcystin-LR) [99], and a series of parabens as personal care products [100]. Another application of these nanophotocatalysts is in the treatment of winery wastewater by a photocatalytic reactor [101]. It has been evidenced in numerous studies that properties such as surface adsorption and photocatalytic reactions of nanocrystalline semiconductor particles are different from those of bulk materials owing to the increased reactive surface area. Application of Nano-Sized TiO_2_ has been approved as a component of photocatalytic film covering scalpels [102], surgical masks [103], and catheters [104]. The UV-based disinfection effectiveness of nano-TiO_2_-coated catheters is three times greater than uncoated ones. Another TiO_2_/UV application with similar results has been demonstrated in infected dental implants [105,106]. The TiO_2_/UV process also showed high bactericidal efficiency in orthopedics, and cosmetic surgery in the presence of *S. aureus* on nano-TiO_2_ coated implants, which is a valuable method for decreasing bacterial infection caused by the application of implants in medicine and biomedical fields [107]. Conventional TiO_2_ photocatalysts, however, cannot provide purified and sufficiently safe drinkable water, since water remains toxic following treatment with TiO_2_ nanoparticles. Therefore, the novel three-dimensional (3D) structured TiO_2_ nanophotocatalyst can be replaced with TiO_2_ nanoparticles by which both the safety level and efficiency of purification of the final purified water are preserved. These structures are suitable for environmental and biomedical applications, as they meet the human key safety conditions, according to an in vitro cytotoxicity test of well-purified water by eco-TiO_2_ [13,108]. Various types of TiO_2_ photocatalytic degradation of organic and inorganic pollutants are summarized in Table 2.

2. Zinc Oxide (ZnO)

ZnO shows efficient activity in photocatalytic degradation of organic contaminants as compared to TiO_2_ [109]. Among different forms of zinc oxides (i.e. ZnO and ZnO_2_), ZnO can form stable, protective coatings, which act as smart materials. Three different crystalline phases of ZnO include zinc-blende, wurtzite, and rock-salt. Due to these multi-functional qualities, ZnO is extensively used for various applications including photocatalysis [110], light-emitting diodes [111], biosensors, solar-cells [112], field-emission and gas sensing [113]. Different morphologies of ZnO have been reported, such as nanorods [114], nanonails, nanopencils [115,116], nanowires [117], nanotubes, nanobullets [118,119], nanocomb-like structures, nanobelts [120], nanoribbons [121,122,123], nanohelices [123], nanoneedles [124], and nanopins [125]. It has been proved that the properties of these materials are strongly dependent on the size and shape of the ZnO nanoparticles. The effective photocatalytic degradation of acridine orange up to about 90% after 80 min of exposure to UV light by ZnO nanocapsule is reported [126,127]. Photocatalytic degradation of methyl orange using ZnO as the photocatalyst has also been achieved—about 99.7% removal of the azo dye in 180 min [128]. In another study, almost complete degradation of methylene blue was obtained within 85 min of irradiation time using ZnO nanoparticles synthesized by hydrothermal treatment [129]. Additionally, ZnO-CeO_2_ nanoparticles, which are discussed in the binary metal oxide category, have been applied to remove methylene blue and acridine orange [130].

3. Iron Oxide (Fe_2_O_3_)

Iron oxides play an important role in many biological and geological processes. They are increasingly applied as pigments, iron ores, catalysts, and as hemoglobin in the blood. Freely dispersed bulk-, sonic-, and nano-Fe_2_O_3_ have been used for photocatalytic oxidation of water under visible and UV irradiation [131]. Iron oxide (α-Fe_2_O_3_) exhibits desirable efficiency as an important photocatalyst [132], with low cost, simple preparation, and n-type semiconducting behavior [133] with no secondary pollution [132]. Due to its various applications as sensors, pigments, actuators, and catalysts, it has attracted considerable attention in recent studies [134,135,136]. Photocatalytic degradation of organic pollutants via Fe_2_O_3_ has been investigated. However, the corresponding photocatalytic mechanism has not been described in detail. The valence electrons of Fe_2_O_3_ compared to those of TiO_2_ can be excited to the conduction band at wavelengths shorter than 560 nm, which can extensively enhance the efficiency of the sunlight use. The maximum degradation efficiency of 94% for dibutyl phthalate in wastewater (as an excellent plasticizer in different resins, especially nitrocellulose and resins and also an vital additive in special paints and adhesives with about 20 years of hydrolysis half-life) was obtained using Fe_2_O_3_ in a photocatalytic process [137]. Compared to α-Fe_2_O_3_ powders, porous α-Fe_2_O_3_ films exhibit better photocatalytic activity by water splitting under UV radiation for hydrogen generation [138]. Photocatalytic oxidation of aniline to azobenzene by Fe_2_O_3_ under UV irradiation and natural sunlight in aprotic and protic solvents has also been reported [139].

4. Gadolinium Oxide (Gd_2_O_3_)

The global interest in using rare earth metals is increasing, due to their distinctive magnetic and electronic attributes in the fashioning of interfaces and surfaces compared to common bulk materials. Gadolinia (gadolinium (III) oxide) is the most widely available derivative form of gadolinium, and is a potential contrast agent in magnetic resonance imaging (MRI). The Gd_2_O_3_-modified bismuth vanadate (BiVO_4_) composite, as a photocatalyst, exhibits significantly greater visible-light photocatalytic activity than pure BiVO_4_ for methyl orange degradation under visible light irradiation [140]. Gd_2_O_3_ nanorods used to detect ethanol by facile hydrothermal routes demonstrated a lower detection limit with higher sensitivity and shorter response time [141] compared to the annealed Gd_2_O_3_ nanostructures [142]. Moreover, a moderate photocatalytic activity was evaluated for degradation of methyl orange by uniform Gd_2_O_3_ hollow microspheres [143]. The degradation of about 90% of 4-chlorophenol using modified Gd_2_O_3_ photocatalyst prepared by the sol–gel method was measured after 4 h of UV light irradiation [144]. In another study, a challenging photocatalyst of Gd_2_O_3_ nanorods was designed for the degradation of neurotoxic chloramphenicol drugs [145].

5. Antimony Oxide (Sb_2_O_4_)

Antimony oxide is classified based on its oxidation states, Sb (III) and Sb (V). Antimony has been applied as a pacifier in enamel, flame retardants, paint and glass art crafts and for making bullets and bullet tracers. It has been used as an alloy for the synthesis of plain bearings, batteries, and solders, as well as as a stabilizer and a catalyst for the preparation of polyethylene terephthalate.

The photocatalytic activity of α-Sb_2_O_4_ has been demonstrated, with almost 52% degradation of acridine orange in 170 min, with low detection limit, good sensitivity, long linear dynamic range with good linearity in a very short response time [146], as well as for the removal of heavy metals (e.g., mercury) from waste water [147], while it has also been reported that synthetic Uranyl Selective Polymeric Membrane sensors based on p-tert-butylbiscalix4arene can be used for the determination of Thorium [148]. The unique characteristics of nanostructures, such as their large surface area, excellent adsorbing and absorbing activity, bio-friendly nature, and high electron exchange could be reasons for the good sensitivity of these systems [149].

##### Binary Metal Oxides

In addition to metal oxides, some other metal oxides have also been studied previously for use in the field of photocatalysis, because of their unique benefits and wide range of applications as catalysts, semiconductors, superconductors, ceramics, antifungal agents, adsorbents, and their specific applications in medicines. Many metal oxide semiconductors (e.g., WO_3_, ZrO_2_, ZnO, and Fe_2_O_3_) that have been exploited in photocatalysts for the degradation of organic contaminants have inherent drawbacks [150]. For example, WO_3_ is a stable photocatalyst for O_2_ production within the visible light irradiation range. However, it is not suitable for H_2_ evolution because of its low level of conduction band. Additionally, α-Fe_2_O_3_ is somewhat stable in acidic solutions, but has the same problems as WO_3_. Moreover, ZnO can be easily corroded under band gap irradiation by photogenerated holes.

**Figure 7:**
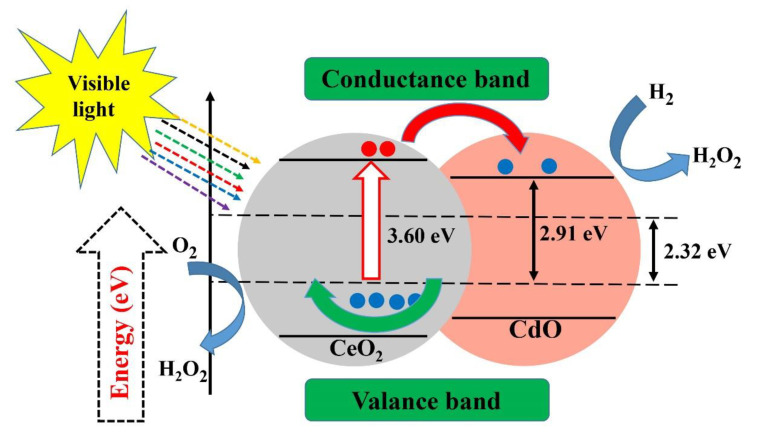
Photocatalytic activity of cerium oxide-cadmium oxide nanocomposites. Reproduced with some modifications from [154].

Ta_2_O_5_ photocatalyst with a nanocrystalline mesoporous structure has recently been synthesized for the production of H_2_ via a sol–gel process combined with a surfactant-assisted templating mechanism [151]. Recently, the effect of Fe-doped NiO as a co-catalyst has also been reported [152]. Additionally, ZnO-CeO_2_ nanoparticles synthesized using an efficient and simple low-temperature method have been successfully applied as photocatalysts for the removal of biomedical and environmental contaminants and reported 80.7% and 92.1% degradation for methylene blue and acridine orange within 170 min of irradiation time, respectively [130]. The Cu_x_S-TiO_2_ composites has shown good efficiency in photo degradation of dyes even under visible light irradiation [153]. In another report, the photocatalytic activity of high-quality CeO_2_-CdO binary metal oxide nanocomposites was evaluated, showing acceptable growth inhibition of *P. aeruginosa* (Figure 7) [154].

##### Metal Sulfides

Metal sulfides have been widely used as visible light responsive photocatalysts. Compared to metal oxides, 3p orbitals of sulfur in their valence band result in a more occupied valence band and a narrower band gap. Recently, among other metal sulfides, ZnS and CdS have attracted great attention. CdS is commonly used for visible light-assisted water splitting due to its suitable band position and band gap (2.4 eV). However, photo-corrosion, which is a common issue in most metal sulfide photocatalysts, occurs when using both CdS and ZnS. Therefore, recent studies have focused on the development of ZnS and CdS photocatalysts, mostly through four different means of improvement: matrixing and supporting the structures of CdS, adding cocatalysts to CdS, preparing porous and one-dimensional CdS, and doping solid solutions of CdS and ZnS [155].

To synthesize porous CdS, a solvothermal method has been used to synthesize CdS nanowires [156] and nanorods [157]. Additionally, mesoporous CdS nanoparticles have been synthesized via ultrasonic and template-free precipitation at room temperature [158]. Nanoporous CdS have also been prepared by including hollow nanorods and nanosheets with 3 nm diameter of pores through a two-step aqueous method [159]. Additionally, CdS quantum dots have been recently loaded on porous polysaccharides and applied as highly efficient contrast imaging agents [160]. On the other hand, due to having an extremely broad band gap to respond to visible light (3.6 eV), solid solutions of ZnS are formed in which the narrow band gap can increase the use of ZnS in visible light. Both CdS and ZnS have the same crystal structures, making it easy to form solid solutions of them [161].

##### Magnetic Nanophotocatalysts

**Figure 8:**
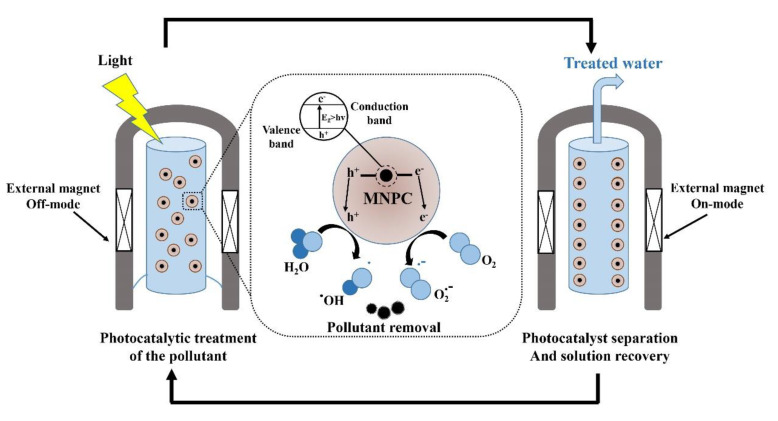
Schematic of the implementation of magnetic nanophotocatalysts (MNPCs) in water treatment. Reproduced from [173].

The incorporation of magnetic nanophotocatalysts in contaminant removal strategies has recently received significant attention due to their improved chemical and physical properties. Therefore, cost-effective, efficient, and environmentally friendly disinfection processes can be achieved due to their easy separation using an external magnetic field, which allows recycling and multiple use of the nanophotocatalyst [162]. They mostly have a core–shell structure consisting of a magnetic core (e.g., iron, cobalt, nickel, and their oxides like maghemite (α-Fe_2_O_3_), magnetite (Fe_3_O_4_), cobalt ferrite (CoFe_2_O_4_)) and a photocatalytic shell (e.g., TiO_2_, ZnO, AgBr, BiOCl) [163]. Furthermore, some nanoferrites like ZnFe_2_O_4_ have shown desirable degradation efficiency of organic target compounds under both visible light and UV irradiation [164]. Similar studies have reported degradation of different contaminants using Fe_3_O_4_ [165,166,167], NiFe_2_O_4_ [168], CoFe_2_O_4_ [169], ZnFe_2_O_4_ [170], BaFe_12_O_19_ [171], SrFe_12_O_19_ [172] -doped TiO_2_ nanophotocatalysts (Table 3). A schematic of the use of magnetic nanophotocatalysts (MNPCs) in water treatment [173] is illustrated in Figure 8.

##### Graphene

**Figure 9:**
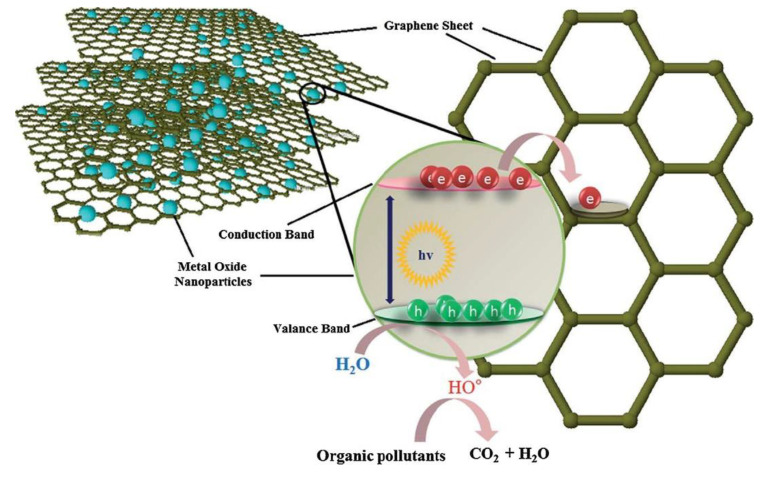
The electron-scavenging nature of graphene from the conduction band of metal oxide [26].

Graphene (G), due to its one-of-a-kind nanostructure and particular properties has been studied widely from both experimental and theoretical scientific points of view [208]. It has already shown promising applications in nanocomposites, nanoelectronics, optoelectronics, drug delivery systems, electrochemical super-capacitors, transistors, solar cells, and chemical sensors (e.g., biosensors, gas sensors, pH sensors) [209]. As shown in Figure 9, graphene has been employed to enhance photocatalytic efficiency, due to its electron scavenging nature, in the conduction band of metal oxide [26]. Some of its novel applications include ultrasensitive high-adsorption ability for various types of contaminations, including arsenic in drinking water [210], brackish water desalination and drinking water purification [211], metal removal from the contaminated environment [212], detection of biomarkers [213], electrochemical sensor for paracetamol [214], treatment of thrombosis [215], protection of DNA from cleavage and its effective cellular delivery [216], MRI and localized photothermal therapy for cancer cell treatment [217], electrochemical immunosensor for sensitive detection of carbohydrate antigen 1.5-3 (CA 15-3) [218], and photothermal agents in NIR region [219]. Modified graphene nanostructures such as P25–G [174,175], TiO_2_–G [176,177,178,179], SnO_2_–G [180], Bi_2_WO_6_–G [181], ZnO–G [182], ZnFe_2_O_4_–G [183], BiVO_4_–G [184], CdS–G [185] have also been reported to have different photodegradation applications (Table 3).

##### Quantum Dots

**Figure 10:**
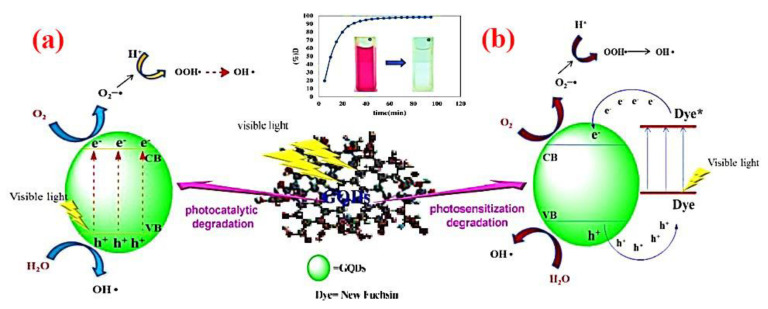
Degradation mechanism of new fuchsin via (**a**) photocatalytic and (**b**) photosensitization pathways using GQDs under visible light irradiation. Reproduced form [192].

Quantum dots (QDs), as zero-dimensional semiconductor multifunctional nanomaterials have been receiving significant attention for the degradation of pollutants [220]. Since QDs have the advantage of the wide band gap of a semiconductor material, they have a promising application as photocatalysts, owing to the swift generation of electron–hole pairs through photoexcitation [204]. On the other hand, photocatalytic, chemical and optical properties of QDs can be improved by surface modification, which also improves the photostability of QDs, as well as the efficacy of light-induced reactions on the QD surface and the generation of new traps on the QD surface [221]. For example, in the self-photosensitization pathway of fuchsin dye degradation, photodegradation can be initiated in the presence of graphene quantum dots (GQDs) under visible light irradiation, as demonstrated in Figure 10 [222]. The application of modified QDs as a photocatalytic agent to degrade pollutants is illustrated in Table 3.

##### Smart Materials (Self-Cleaning)

Smart photocatalytic materials have been developed widely over the past two decades [49]. Different kinds of applications such as simultaneous self-cleaning and air cleaning have mostly focused on the use of TiO_2_ and ZnO due to their low cost, high stability and strong capacity for the photocatalytic decomposition of organic contaminants [205]. TiO_2_ has been used recently to make self-decontaminant textiles that offer high antibacterial activity performance for UV shielding [206]. Nanophotocatalysts can be merged onto different surfaces of bulk structures (i.e. concrete) [223] or onto the glass of windows, flat surfaces, or walls [224].

**Figure 11:**
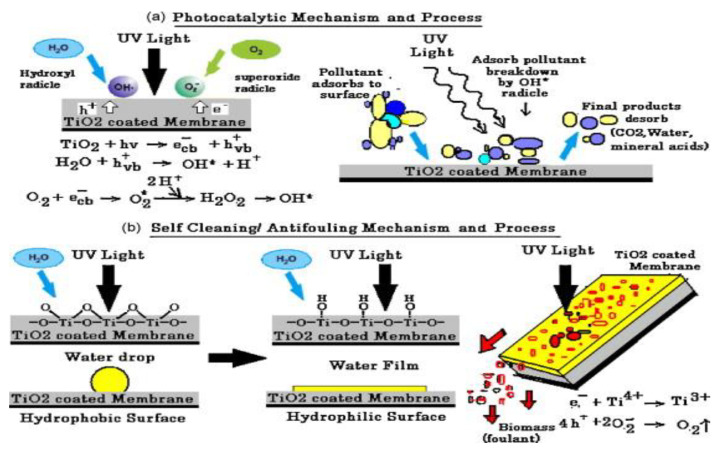
(**a**) Photocatalysis mechanism and process. (**b**) Self-Cleaning/antifouling mechanism and process of polyvinylidene fluoride (PVDF)/TiO_2_ membrane. Reproduced form [207].

TiO_2_-coated membranes offer outstanding antifouling/self-cleaning, photoactive, and bactericidal properties that are based on the UV mechanism of TiO_2_ photocatalysis (Figure 11) [207]. The overall applications of different types of nanophotocatalysts mentioned in this review are summarized in Table 3.

## 5. Future Prospects, and Concluding Remarks

Biomedical waste eradication strategies are still very limited due to low photocatalytic efficacy. Therefore, extensive assessments are highly demanded from the practical point of view [225]. The applications of TiO_2_ photocatalysts, for example, is restricted because of poor quantum efficacy as a result of limited absorption within only UV range (4% of sunlight). The recent advancement in the field of photocatalysis technology is investigating novel agents with higher photocatalytic performance to expand their light respond range. To address these issues, coupled semiconductor, noble metal deposition and ion modification, are the proposed methods to improve energy band and photocatalytic efficacy of explicit applications. In addition, due to concurrent photocatalytic and redox reactions, sensible photocatalytic systems can be designed for the simultaneously photocatalytic treatment of two or more contaminants [226]. However, several issues (e.g., evaluating the immobilization of photocatalysts and suspension systems) should be contemplated for further advancement. Solar-Based photocatalytic methods have shown better performance than conventional methods in the removal of tenacious organic contaminations [227]. To improve the photodegradation of wastewater, suitable surface modification technique of photocatalysts is an essential need. In addition, development of nanostructures, photoactivity of recycled photocatalysts, mechanisms of degradation, recovery of photocatalyst during treatment, and interactions between the photocatalysts and the pollutants are yet to be further improvements. To predict the kinetics, quantum yield and optimized conditions of the process, further investigations are required to verify the mathematical models for photocatalytic systems. The future improvement of nanophotocatalysts would be made by making them multifunctional and controllable enough to be subsequently transformed into nano-gadgets. To facilitate the accessibility of these innovations, extensive endeavors are expected to defeat the challenges in the future.

The implementation of the photocatalytic method for disinfection and cleansing is an adaptable and effective procedure for incapacitating a broad range of adverse microorganisms in different media. This approach is a non-toxic, safe, and cost-effective sterilization technique whose versatility enables it to be used in various purposes. Nanotechnology has shown an incredible potential to improve the effectiveness of biomedical waste treatment [228]. Therefore, the use of nanophotocatalysts has become of great interest in biomedical waste management as they can provide excellent and practical applicability. Improving the biomedical waste management; however, should start with the reduction of wastes production according to the norms, rules, and standards in each country, with respect to regulation of biomedical waste disposal in various categories. Besides, practicing the optimized models for monitoring the waste produced by hospitals, health-care centers as well as research into eco-friendly sustainable technologies, recycling and PVC-free devices will go in a long way for a safe environment. Globally, more focused research in the field of biomedical waste management required to comprehend its impact on the field of public health better.

There is an ongoing research in field of nanomaterials to design and develop nanophotocatalytic reactors. Despot of the advancements in field of nanophotocatalytic materials, further investigations are required to be done to characteristics the nanophotocatalytic materials. The major remaining challenges are strengthening the process, mass transfer limitations and high photons consumption. Therefore, the concept of using nanocomposites is ideal to resolve the issues related to electron pair recombination which can be prolonged by combining the nanocomposites with nanophotocatalytic reactor structures. The recent reactors known as microfluidic reactors open a new opportunity for intense characteristics study in reaction and synthesis phase. Microfluidic reactors are based on micro level reactants. The remarkable features of these reactors are; improved diffusion effect and great mass transfer coefficient factor, large surface-to-volume ratio, highly stable hydrodynamics, less Reynold’s flow, and informal handling which make them better candidate compared to the conventional reactors. However, implementing the photocatalysis in a larger scale and actual wastewater is still challenging. Synthesis of structures such as nanorod, nanosphere, nanoflowers, nanoflakes and nanocones with improved functional and structural properties could open a new area of study in this subject. Crucially nanophotocatalysts with excellent efficiency, inexpensive, eco-friendly and high stability are needed to be synthesized [229].

## 6. Importance of Waste Management as a Crucial Public Service during the COVID-19 Outbreak

Since the outbreak of the coronavirus (COVID-19), its impact upon human health and the economy has been increasing day by day; administrations are advised to treat waste management, including of medical, household and other harmful waste, as an urgent and crucial public service in order to reduce the possible risk of secondary impacts upon public health and the environment.

During such an epidemic, various types of medical and hazardous waste are generated, including infected personal protective equipment (PPE) masks, gloves and other protective equipment, as well as a higher volume of non-infected items of the same nature that can easily become mixed with domestic garbage, but should be treated as hazardous waste and disposed of separately from other household waste streams. Unreliable waste management could lead to unexpected “knock-on” impacts on human health and the environment. During the COVID-19 emergency, safe management of household waste is also expected to be critical. To overcome this enormous and unprecedented challenge, decision-makers are urged to make every effort to ensure that waste management, including that from medical and household sources, is given the attention—indeed priority—it requires in order to minimize its impact upon human health and the environment.

The COVID-19 outbreak has made a global demand to effectively diagnose, treat and mitigate the spread of the infection, through comprehensive approaches such as specific alternative antiviral methods and classical disinfection protocols. The physicochemical properties of materials can be engineered to offer distinctive approaches to manage this emergency. Considering the life cycle of the virus, it is envisioned that nanotechnology could be employed to encounter the disease; nanoparticles (NPs), for instance, can be used as an alternative to the conventional disinfection protocols used in the healthcare system, owing to their inherent antipathogenic properties and their ability to inactivate viruses, bacteria, fungi, and yeasts either through photothermal or generation of photocatalysis-induced reactive oxygen species (ROS). In conclusion, nanotechnology can play a critical role in counteracting COVID-19 and preparing for future pandemics [230].

## Figures and Tables

**Table 1:** Summary of the main findings of nanophotocatalytic investigations.

Nanophotocatalyst	Contaminant Conversion	Operative Conditions	Duration/Performance of Nanophotocatalyst	By-Products	Ref.
TiO_2_@Plexiglas	*E. coli*, *P. aeruginosa*, *S. aureus* and *E. faecium*	Photocatalysis on TiO_2_-coated Plexiglas with indirect UVA light irradiation (60 min) with and without time resolution	Reduction efficiency more than 6 log10 steps in 60 min in disinfecting the surfaces	Oxygen radicals and hydrogen peroxide	[53]
TTIP and TiCl_4_/TiO_2_ films@stainless steel and @SiO_2_ layers	Destruction of stearic acid layers	Photocatalytic activities of TTIP and TiCl_4_ TiO_2_ films (156 nm) deposited onto stainless steel, silica (30 nm) and (120 nm) under 365 nm UV irradiation	Photocatalytic activity of 7.6 × 10^−4^ and 9.0 × 10^−5^ cm^−1^ min^−1^ for the TTIP and TiCl_4_-grown films, respectively	Iron and chromium	[54]
TiO_2_ PC105 (15–25 nm with a nanoanatase phase of 100%)	Inactivation of *E. coli*	Irradiation with fluorescent light produced by eight 8 W lamps with visible light and UV (290–400 nm) at 0.05–0.12 Wm^−2^ intensity	With loadings ranging from 520 to 15,590 mgm^−2^, *E. coli* inactivation as a function of time was monitored for up to 120 min	Hydroxyl and hydroperoxyl radicals	[56]
TiO_2_ thin films on stainless steel	Destruction of stearic acid layers, monitored using FT-IR spectroscopy	Pre-Activation of the sample using UVA (365 nm) radiation (2.24 mW cm^−2^, 24 h), exposure to the same UVA radiation for timed intervals	Biocidally effective against *E. coli* with a 100% kill (6 log reduction) in less than 3 h	HCl	[57]
Transparent TiO_2_ films on stainless steel	Oxidation of acetone in air at ambient temperature using a 7000 mL reactor	UV illumination by a 15 W 365 nm UV lamp with a UV intensity of 540 (10 íW/cm^2^ in an ambient condition (22 °C, RH 80%, in air)	Excellent photoinduced hydrophilicity and antibacterial effect for *Bacillus pumilus* on the TiO_2_ films with 3 h calcination under UV illumination decreases to 50% within 2 h	Fe^3+^ ion, carbon dioxide	[58]
Copper-Deposited TiO_2_ Thin Film	Two types of *E. coli* cells (IFO 3301 strain and 53TNE007 strain)	Fluorescence intensities at 460 nm under various conditions were compared to that observed from the TiO_2_ film after UV light illumination with intensity of 1 mW/cm^2^ for 20 min.	Efficient bactericidal function for various bacteria compared to the conventional copper system in an ordinary living space with very weak UV intensity	Cu^+^, hydroxyl radicals, 7-hydroxycoumarin	[59]
Nitrogen-Doped TiO_2_ substrates	Pathogens, including *Shigella flexneri*, *Listeria monocytogenes*, *Vibrio* parahaemolyticus, *S. aureus*, *Streptococcus pyogenes*, and *Acinetobacter baumannii*	Visible light illumination under the incandescent lamp for 5 min at 4 °C at a distance of 5 cm, corresponding to an illumination density of 3 × 10^4^ lux.	Superior visible-light-induced bactericidal activity against *E. coli* compared to pure TiO_2_ and carbon-doped TiO_2_ substrates	ROS, exotoxin	[60]
N, S co-doped commercial TiO_2_ powders (Tayca TKP101, TKP102)	*E. coli*	Illumination during 2 h and samples (1.0 mL) were taken at different time intervals.	Suitable photocatalytic activity under UV illumination towards *E. coli* inactivation and also under visible light irradiation (400–500 nm)	ROS, inactivate bacterial cells, pyrosulfate, SO_4_^2−^ and SO_2_	[61]
N, S co-doped TiO_2_ (Tayca)	Phenol, Dichloroacetate, *E. coli*	Inactivation were achieved under UV illumination (pH 6.0, UV intensity 30 Wm^−2^ and concentration of N, S TiO_2_ of 1 g L^−1^) and visible light irradiation (400–500 nm) for 16 min	Strong photocatalytic activity towards the photo-degradation of phenol and dichloroacetate, and inactivation of *E. coli* under exposure to UV light	Hydroxyl and O_2_^−^ radicals, ^1^O_2_, p-benzoquinone	[62]
N, S co-doped and N-doped Degussa P-25 powders	*E. coli* inactivation and phenol oxidation	Photocatalytic activities of the powders were tested using phenol and *E. coli* cells under UV intensity of 38 W m^−2^ (320–380 nm) and visible light (400–500 nm)	The highest photocatalytic *E. coli* inactivation under visible light with Degussa P-25	P-benzoquinone, Hydroxyl and O_2_^−^ radicals, ^1^O_2_	[63]
TiO_2_ (P25 formulation; Degussa)	Inactivation of *Legionella pneumophila*	Inactivation of the viability of selected L. pneumophila strains and controls (with initial cell concentration of 107 cfu/mL) at different time intervals of PCO using 1000 mg/L of TiO_2_ and 108 µW/cm^2^ of UV365 nm	Total mineralization of bacterial cell with prolonged photocatalytic oxidation treatment with the highest inactivation efficiency (IE, log-reduction) after 90 min	OH radicals	[64]
TiO_2_ plasma sprayed coating on stainless steel 304	Methylene blue aqueous solution decomposition	Irradiation using ultraviolet rays (390 nm) lamp which excites electrons and forms holes in TiO_2_ coatings	A lower heat input resulted in a higher anatase phase fraction and smaller anatase grain size and the best photodecomposition efficiency	Superoxide ions and hydroxyl radicals	[65]
TiO_2_ films electrolytically deposited on AISI 316L stainless steel and Ti_6_Al_4_V substrates	Vanadium, aluminum, sulfur and phosphorus	Scratch tests on electrolytic TiO2 deposited −75 mA cm^−2^/8 C on AISI 316 L after annealing	Excellent adhesion and very ductile behavior were found from nanoindentation and scratch tests	Fe (3–4 at. %) and Cr (1 at. %), peroxo-complex	[66]
Needle-Like shaped uniform anatase TiO_2_ coatings on MWNTs	Bacterial endospores (*Bacillus cereus*)	UV lamps were stabilized for 30 min to obtain constant intensity (92 W/m^2^) before each test	90% inactivation of spores (LD90) and also in terms of time required to achieve a 1.0 log10 reduction of spores in the tail region of the inactivation curve	Hydroxyl radicals	[67]
Sulfur-Doped and Nitrogen-Fluorine-codoped TiO_2_	Photoinactivation of *E. coli*	Under solar simulated light (UVA 3 mW/cm^2^; 162,370 lx) and visible light (162,370 lx) irradiation for 30 min	S-TiO_2_ photocatalysts did not show any enhancement in photocatalytic activity toward *E. coli* inactivation under visible light irradiation	ROS, OH and O_2_^−^ radicals	[68]
Ceramic tiles coated with TiO_2_	*Salmonella Enteritidis*	Radiation of UV-C of 253.7 nm wavelength for 0, 60, 90, and 120 s.	Bactericidal action of UV radiation is much stronger on the surfaces of TiO_2_-coated tiles than on the uncovered tiles	pyrimidine dimers	[69]

**Table 2:** TiO_2_ photocatalytic degradation of organic and inorganic pollutants. With some modifications from [13].

Type		Pollutant	Catalyst
Organic pollutants	Dye wastewater	Methyl orange	Y-TiO_2_-HPW
		Alkaline red dye	TiO_2_-Fenton
		Rhodamine 6G	TiO_2_
		Anthraquinone dye	N-TiO_2_
	Pharmaceutical wastewater	Amoxicillin, Penbritin	TiO_2_
		Cloxacillin, Oxolinic acid	TiO_2_
	Pesticide wastewater	Kappa furan pesticides	TiO_2_
		Armour mix phosphorus	TiO_2_
		Alon	TiO_2_-SBA
		Organophosphorus pesticide	TiO_2_
	Explosives wastewater	TNT, RDX, HMX	TiO_2_
	Chlorine hydroxybenzene wastewater	Chlorinated phenol	TiO_2_
	Nitrobenzene wastewater	Nitrobenzene	H_3_PW_12_O_40_/TiO_2_
Inorganic pollutants	Heavy metal pollutants	Hg (II)	TiO_2_
		Cr (VI)	ZrO_2_
		Mn (II), Ti (I)	TiO_2_
	Cyanide-containing waste	CN^−^	TiO_2_
	NO^−^_2_ containing waste	NO^−^_2_	Fe^3+^/TiO_2_/SiO_2_

**Table 3:** The summary of typical applications of different types of nanophotocatalysts.

Type of Nanophotocatalyst		Photocatalytic Activity	Ref (s)
Metal oxides	TiO_2_	Degradation of expired drugs and pharmaceutical compounds, dyes in textile industries, pesticides, cyanobacterial toxin microcystin-LR, parabens. Photocatalytic films covering scalpels, surgical masks, and catheters	[96,102]
	ZnO	Photocatalytic degradation of acridine orange, methyl orange (MO), methylene blue (MB)	[126,127]
	Fe_2_O_3_	Photodegradation of dibutyl phthalate in wastewater, Photocatalytic oxidation of aniline to azobenzene	[138,140]
	Gd_2_O_3_	Photodegradation of MO, 4-chlorophenol, neurotoxicity chloramphenicol drug	[144]
	Sb_2_O_4_	Photodegradation of acridine orange, Removal of heavy metals (e.g., mercury) from wastewater	[147]
Binary metal oxides	ZnO-CeO_2_	Photodegradation for MB and acridine orange	[130]
	Cu_x_S-TiO_2_	Photodegradation of dyes	[154]
	CeO_2_-CdO	Antimicrobial activity of bacteria and fungi	[155]
Metal sulfides	ZnS, CdS	Visible light assisted water splitting	[156]
Magnetic nanophotocatalysts	Fe_3_O_4_@TiO_2_	Degradation of rhodamine B (RhB), MB, Quinoline	[166,167,168]
	NiFe_2_O_4_@TiO_2_	Degradation of MO	[169]
	CoFe_2_O_4_@TiO_2_	Degradation of procion red MX-5B (PR)	[170]
	ZnFe_2_O_4_@TiO_2_	Degradation of MO	[171]
	BaFe_12_O_19_@TiO_2_	Degradation of PR	[172]
	SrFe_12_O_19_@TiO_2_	Degradation of PR	[173]
Graphene	P25–G	Decomposing MB under UV and visible light, Decomposing benzene (gas phase) under UV light	[174,175]
	TiO_2_–G	Decomposing MB under sunlight light, Decomposing MB under UV light, Decomposing MO under UV light, Decomposing rhodamine B (RhB) under UV light, Decomposing RhB under visible light	[176,177,178,179]
	SnO_2_–G	Decomposing RhB under visible light	[180]
	Bi_2_WO_6_–G	Decomposing RhB under visible light	[181]
	ZnO–G	Decomposing MB under UV light	[182]
	ZnFe_2_O_4_–G	Decomposing MB under visible light	[183]
	BiVO_4_–G	Photoelectrochemical water splitting	[184]
	CdS–G	Photocatalytic H2 evolution under visible light	[185]
Quantum dots	ZnS QDs	Degradation of Methyl violet, Victoria blue, Malachite green, Thymol blue, Congo red, Safranin, MB, HMX (octahydro-1,3,5,7-tetranitro-1,3,5,7-tetrazocine), RDX (hexahydro-1,3,5- trinitro-1,3,5-triazine)	[186,187,188,189,190,191,192,193,194,195,196,197,198,199,200,201,202,203,204]
	Graphene QDs	Degradation of New fuchsin, RhB, MO	[192,193,194]
	Carbon QDs/BiOX (X = Br, Cl)	Degradation of Phenol RhB, Ciprofloxacin, Bisphenol A (BPA)	[195]
	Carbon QD/NZnO	Degradation of Malachite green, MB, Fluorescein	[196]
	Graphitic carbon nitride QDs	Degradation of RhB	[197]
	CdS QDs	Degradation of Alizarin, Acid violet, Mordant red, Thymol blue	[198]
	CdSe/ZnS QDs	Degradation of Methyl green	[199]
	TiO_2_ QDs	Degradation of Indigo carmine, Ketorolac tromethamine	[200,201]
	ZnO foam/carbon QDs	Degradation of RhB, MO, MB	[202]
	Ag@AgCl QDs Sensitized Bi_2_WO_6_	Degradation of RhB	[203]
Smart materials (self-cleaning)	ZnO	Decomposition of organic contaminants	[205]
	TiO_2_	Self-decontamination textiles, the antibacterial activity of UV shielding	[206]
	PVDF/TiO_2_	Antifouling/self-cleaning, photoactive, and bactericidal	[207]
